# A Bayesian Network Driven Approach to Model the Transcriptional Response to Nitric Oxide in *Saccharomyces cerevisiae*

**DOI:** 10.1371/journal.pone.0000094

**Published:** 2006-12-20

**Authors:** Jingchun Zhu, Ashwini Jambhekar, Aaron Sarver, Joseph DeRisi

**Affiliations:** 1 Department of Biochemistry and Biophysics, University of California San Francisco, San Francisco, California, United States of America; 2 Howard Hughes Medical Institute, University of California San Francisco, San Francisco, California, United States of America; IBM Thomas J. Watson Research Center, United States of America

## Abstract

The transcriptional response to exogenously supplied nitric oxide in *Saccharomyces cerevisiae* was modeled using an integrated framework of Bayesian network learning and experimental feedback. A Bayesian network learning algorithm was used to generate network models of transcriptional output, followed by model verification and revision through experimentation. Using this framework, we generated a network model of the yeast transcriptional response to nitric oxide and a panel of other environmental signals. We discovered two environmental triggers, the diauxic shift and glucose repression, that affected the observed transcriptional profile. The computational method predicted the transcriptional control of yeast flavohemoglobin *YHB1* by glucose repression, which was subsequently experimentally verified. A freely available software application, ExpressionNet, was developed to derive Bayesian network models from a combination of gene expression profile clusters, genetic information and experimental conditions.

## Introduction

Nitric oxide (NO·) is a critical mediator of the cell's innate immune response that defends against infection caused by a wide range of pathogens including fungi, bacteria, protozoan parasites and viruses [1]–[5]. Thus, a critical biological counter-measure would include the ability to detoxify or limit the damage from NO·. However, the mechanism by which pathogens defend against nitric oxide is not well understood. Recently, genomic surveys of NO· triggered transcriptional responses have been carried out in several fungal organisms (*S. cerevisiae, H. capsulatum*, and *C. albicans*) using microarrays [6]–[8]. In *S. cerevisiae*, five genes were identified as the NO· detoxification gene cluster, which are activated through the transcription factor Fzf1p when exposed to exogenously supplied NO·. The five genes are the yeast flavohemoglobin *YHB1*, whose *E. coli* homolog has been shown to convert NO· to nitrate as a potential mechanism for NO· detoxification, a putative sulfite pump *SSU1*, and three additional uncharacterized open reading frames [8].

In addition to the detoxification gene cluster activated through Fzf1p, the microarray data also revealed alterations of mRNA abundance for many other genes during NO· treatment [8] including genes involved in the yeast environmental stress response [9]. The pattern of yeast expression profiles was further compounded by the usage of a variety of genetic mutants and conditions that led to a complex and overlapping series of cellular responses. These include large variations in cell culture density, carbon source, and genotypes. Although a preliminary analysis by simple hierarchical clustering was sufficient to identify the NO· detoxification cluster, we sought to leverage the complexity of the dataset to further dissect additional regulatory mechanisms operating in these experiments.

Bayesian belief networks, a form of graphical probabilistic models encoding dependence relationships among interacting variables as probability distributions, offer a promising computational strategy for elucidating hidden inputs into nitric oxide regulation [10], [11]. A Bayesian network uses nodes to model variables of interest such as gene activation or environmental perturbation. The relationship among variables, such as NO· treatment-triggered gene activation, can be modeled as conditional probability distributions. Given a set of observed data such as a microarray dataset, a probabilistic score can be assigned to each network model. The best models can be derived automatically from experimental data using a network learning procedure [10], [11], and the derived model can be viewed as an interpretation for the biological dataset. Similar Bayesian network-driven approaches have been successfully applied to model signaling networks in primary human immune system cells, identify regulatory modules and their condition-specific regulators in *S. cerevisiae*, decode the combinatorial code underlying gene expression, and infer gene regulatory networks by using expression levels of individual genes as network nodes [12]–[19].

In addition to modeling multivariable systems, a Bayesian network provides a natural platform for incorporating prior biological knowledge. For example, the knowledge of stress-inducible transcriptional responses can help to explain part of the NO· triggered transcription profile. A Bayesian network can incorporate such prior biological knowledge by encoding biological variables such as the presence of stress as network nodes and known biological relationships as probability distributions [20].

The goal of this work was to combine prior biological knowledge, computational modeling, and experimental feedback in an iterative cycle of hypothesis generation and testing to build a network model to decode the relationship of NO·, the transcription factor Fzf1p and other environmental signals using the genome-wide transcriptional output as measured by microarrays. Rather than simply recapitulate what was already known, we sought to develop and use a Bayesian network-driven approach to uncover previously unrecognized mechanisms of control operating within this system. As a result, we discovered two previously unappreciated environmental variables that affect regulation of *YHB1* (flavohemoglobin) mRNA abundance. Through additional experiments, we showed that flavohemoglobin expression, the primary effector of nitric oxide detoxification, is further regulated by glucose repression. Therefore, *YHB1* transcription cannot be viewed as simply constitutive, or even a switch solely controlled by nitric oxide exposure, but rather as receiving a combinatorial input from many signals. As part of this process, we have produced a freely available software package, ExpressionNet, for exploring complex datasets using Bayesian networks.

## Materials and Methods

### Microarray experiments

#### E1: NO· perturbation

Log phase (OD_600_ 1.0) strains were treated with NO· released from 1mM DPTA-NONOate (DBY7283, BY4741 *fzf1*Δ) and NO· gas bubbling through the media for 10 sec (DBY7283). mRNA isolated from treated (DPTA-NONOate exposure for 10, 20, 40, 80, 120 min; 120 min after gas bubbling) or untreated culture (0 min) was used to generate the Cy5 or Cy3 cDNA probes.

#### E2: Glucose to galactose I

DBY7283 strains with plasmids containing either *GAL1p:LacZ* or *GAL1p:FZF1* were grown to OD_600_ 1.0 in SD-URA, washed by water, then transferred to SGal-URA for continuing growth. mRNA isolated from treated (8, 12 hr after the transfer) or untreated culture (0 hr) was used to generate the Cy5 or Cy3 cDNA probes.

#### E3: Glucose to galactose II

Stationary phase (3-day-old saturated glucose culture) DBY7283 and S288c *fzf1Δ* strains were inoculated at OD_600_ 0.5 in SCD, grown for 2 hr, washed by water, then transferred to SCGal for continuing growth. Total RNA isolated from treated (4, 8, 12 hr after the transfer) or untreated cultures (0 hr) was used to generate the Cy5 or Cy3 cDNA probes.

#### E4: Raffinose to galactose

DBY7283 and JZY100 (DBY7283, *FZF1* deleted with KanMX) strains were grown to early log phase in SC raffinose. Galactose was added into the media to a final concentration of 2% for continuing growth. Since galactose is the preferred substrate and raffinose is not known to interfere with induction of the galactose pathway, there is no need to remove the remaining raffinose. mRNA isolated from sample (0, 30, 60, 120, 240 min after adding galactose) or reference (DBY7283; combined 0 and 240 min) culture was used to generate the Cy5 or Cy3 cDNA probes.

Differentially labeled cDNA probes were hybridized to yeast cDNA microarrays containing PCR probes of all yeast genes [22]. Microarray production, RNA isolation, cDNA synthesis, amino-allyl dye coupling, hybridization and data collection were performed as previously described [22]. Microarray data were normalized using the NOMAD database (ucsf-nomad.sourceforge.net). Spots flagged by GenePix® Pro v3 (Axon Instruments) were excluded from analysis. Additional spots excluded from the analysis were both Cy3 and Cy5 signal intensities less than 2 times the background (E1, E2) and with feature intensity less than the background (E3, E4). The E4 dataset was transformed (i.e. normalized) by its 0 min data point. Complete microarray data are available at NCBI Gene expression omnibus database.

### Data source and preprocessing for network learning

#### Initial model

The microarray data included those from experiments E1 or E2 (19 arrays) and the published dataset of yeast treated with H_2_O_2_ or menadione over 0 to 160 min (21 arrays) [9]. A subset of 130 genes with greater than two fold change in three or more data points in the E1 and E2 experiments were selected. The genes were clustered using data from experiments E1 and E2 [24]. Five major gene clusters were identified using correlation cutoff 0.75 with subsequent manual adjustment: Fzf1p early response, Fzf1p late response, ESR, oxidative phosphorylation, and galactose response clusters (Table 1). The manual adjustment consisted of combining galactose up-regulation and down-regulation clusters to the galactose response cluster and splitting the Fzf1p response cluster into Fzf1p early and late response clusters based on their initial response times (5–15 min vs. 15–45 min).

**Table 1:**
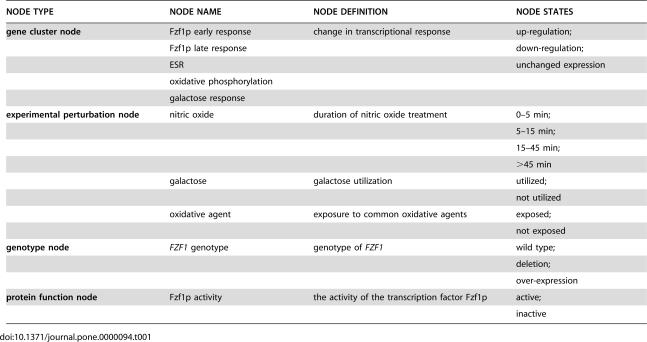
Network nodes defined in the initial nitric oxide response model.

NODE TYPE	NODE NAME	NODE DEFINITION	NODE STATES
**gene cluster node**	Fzf1p early response	change in transcriptional response	up-regulation;
	Fzf1p late response		down-regulation;
	ESR		unchanged expression
	oxidative phosphorylation		
	galactose response		
**experimental perturbation node**	nitric oxide	duration of nitric oxide treatment	0–5 min;
			5–15 min;
			15–45 min;
			>45 min
	galactose	galactose utilization	utilized;
			not utilized
	oxidative agent	exposure to common oxidative agents	exposed;
			not exposed
**genotype node**	*FZF1* genotype	genotype of *FZF1*	wild type;
			deletion;
			over-expression
**protein function node**	Fzf1p activity	the activity of the transcription factor Fzf1p	active;
			inactive

#### Second model

The microarray data included those in the initial modeling plus array data generated from experiment E3 and a published dataset monitoring the transcriptional response of the diauxic shift [22]. The 130 genes selected in the initial modeling were re-clustered using all the above microarray data [24]. Five major gene clusters were identified using cutoff 0.6 with subsequent manual adjustments: ESR, Fzf1p response, YHB1, galactose utilization and energy clusters (Table 2). The energy cluster included genes in the previously designated oxidative phosphorylation cluster and genes in the glucose utilization pathway that were previously in the galactose response cluster. After obtaining the new data of experiment E3, we realized the crucial separation within the Fzf1p response cluster was that between *YHB1* and the rest of the cluster. Therefore, the Fzf1p response clusters were not separated into early and late response clusters as in the initial model. The manual adjustments consisted of separating *YHB1* from the Fzf1p response cluster and forming a separate cluster containing only *YHB1*.

**Table 2 pone-0000094-t002:**
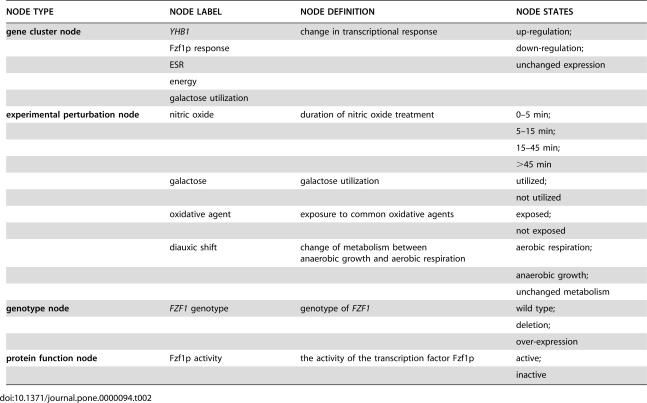
Network nodes defined in the second nitric oxide response model.

NODE TYPE	NODE LABEL	NODE DEFINITION	NODE STATES
**gene cluster node**	*YHB1*	change in transcriptional response	up-regulation;
	Fzf1p response		down-regulation;
	ESR		unchanged expression
	energy		
	galactose utilization		
**experimental perturbation node**	nitric oxide	duration of nitric oxide treatment	0–5 min;
			5–15 min;
			15–45 min;
			>45 min
	galactose	galactose utilization	utilized;
			not utilized
	oxidative agent	exposure to common oxidative agents	exposed;
			not exposed
	diauxic shift	change of metabolism between anaerobic growth and aerobic respiration	aerobic respiration;
			anaerobic growth;
			unchanged metabolism
**genotype node**	*FZF1* genotype	genotype of *FZF1*	wild type;
			deletion;
			over-expression
**protein function node**	Fzf1p activity	the activity of the transcription factor Fzf1p	active;
			inactive

#### Third model

The microarray data included those in the second model as well as those generated from experiment E4. Gene cluster nodes were identical to those defined in the second model.

### Node states and the learning datasets

The discrete states for network nodes in Table 1–3 were defined as the following: Node states for “NO treatment” were defined as the time intervals that best delineated the dynamics of the gene expression response following the treatment. The intervals were as follows: 0–5 minutes (very little transcriptional response), 5–15 minutes (increased transcriptional response), 15–45 minutes (sustained transcriptional changes), and >45 minutes (decreased transcriptional response).

**Table 3:**
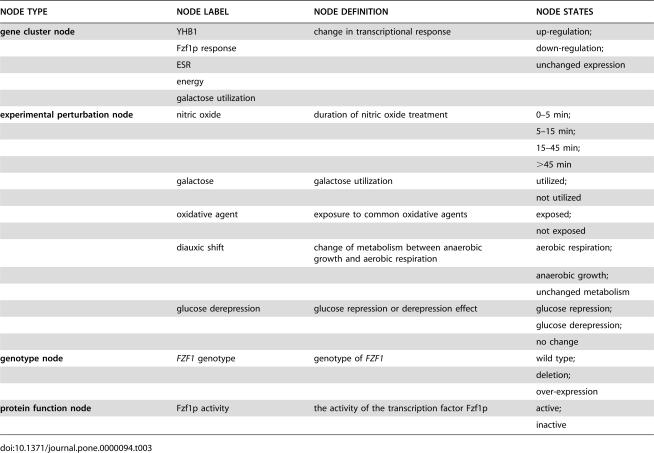
Network nodes defined in the third nitric oxide response model.

NODE TYPE	NODE LABEL	NODE DEFINITION	NODE STATES
**gene cluster node**	YHB1	change in transcriptional response	up-regulation;
	Fzf1p response		down-regulation;
	ESR		unchanged expression
	energy		
	galactose utilization		
**experimental perturbation node**	nitric oxide	duration of nitric oxide treatment	0–5 min;
			5–15 min;
			15–45 min;
			>45 min
	galactose	galactose utilization	utilized;
			not utilized
	oxidative agent	exposure to common oxidative agents	exposed;
			not exposed
	diauxic shift	change of metabolism between anaerobic growth and aerobic respiration	aerobic respiration;
			anaerobic growth;
			unchanged metabolism
	glucose derepression	glucose repression or derepression effect	glucose repression;
			glucose derepression;
			no change
**genotype node**	*FZF1* genotype	genotype of *FZF1*	wild type;
			deletion;
			over-expression
**protein function node**	Fzf1p activity	the activity of the transcription factor Fzf1p	active;
			inactive

States for the gene expression nodes were defined as up-regulation, down-regulation and unchanged expression, using a 2-fold threshold to convert microarray readouts to discrete values.

Node states for “galactose” were defined as “utilized” and “not utilized” depending on whether the substrate was introduced into the media. Similar states were defined for the node “oxidative agent”.

Nodes for “diauxic shift” were defined with three states: aerobic respiration, anaerobic growth and unchanged metabolism; depending on the presence and direction of the shift. Similar node states were defined for the node “glucose derepression”.

The learning datasets were compiled using 1) average gene expression fold change of each gene cluster converted into discrete values using a 2-fold threshold, and 2) manual annotation of the remaining values (experimental perturbations, *FZF1* genotype and Fzf1p activity) based on the experimental conditions and strain genotypes (missing values were allowed). The complete learning datasets and gene membership of each cluster are available at http://derisilab.ucsf.edu/network.

### Bayesian network learning and software implementation

A software application, ExpressionNet, was developed to perform Bayesian network learning. We used a Bayesian scoring function to assign a probability score for a network model. The clique-tree technique and the variable-elimination technique were implemented for efficient inference and learning [11], [20], [25]. The learning process started with random edge combinations, gradually improving the network topology using a greedy search strategy until the score reached a local maximum. The greedy search was iterated to generate a collection of high scoring networks. High scoring networks were subjected to small topology changes by single edge addition, deletion or reversion to expand the collection. Learning was repeated using two different prior probability distributions of the network parameters (priors), both set as a Dirichlet distribution: *Dir*(1,1, …, 1) and *Dir*(P_0_·α, P_0_·α, … , P_0_·α ), where P_0_ is a uniform distribution over the probability space of each CPD and α = 5. Networks scoring within a percentile cutoff (15% for the initial and second models, 25% for the third model) using both priors were used to construct average Bayesian network models. We defined all environmental and genotype nodes as root nodes and all gene cluster nodes as leaf nodes. Missing values were handled using a Structural Expectation-Maximization algorithm [26]. ExpressionNet is available at http://expressionnet.sourceforge.net/. The derived network models and probability parameters are available at http://derisilab.ucsf.edu/network.

### Flow cytometry

The *YHB1-GFP* strain is a C-terminus fusion of GFP obtained from a genome-wide tagged library [27]. The culture was grown to early log phase in synthetic media with 2% glucose, raffinose or galactose, washed with PBS, then transferred to synthetic medium with 2% glucose (from raffinose or galactose), or raffinose or galactose (from glucose). The cell fluorescence intensities were measured on a Becton Dickinson LSR II flow cytometer at 0, 2, 4.5, 6, 8.25, and 12 hr after the sugar was changed. For each time point, a minimum of 100,000 cells were measured to derive the mean GFP intensity. *TUP1* was deleted with KanMX in the *YHB1-GFP* strain. Identical experiments were performed as described earlier. Cell fluorescence intensities were measured at 0, 2, 4, 6, and 18 hr after the sugar was changed.

## Results

### Algorithms

**Figure 1:**
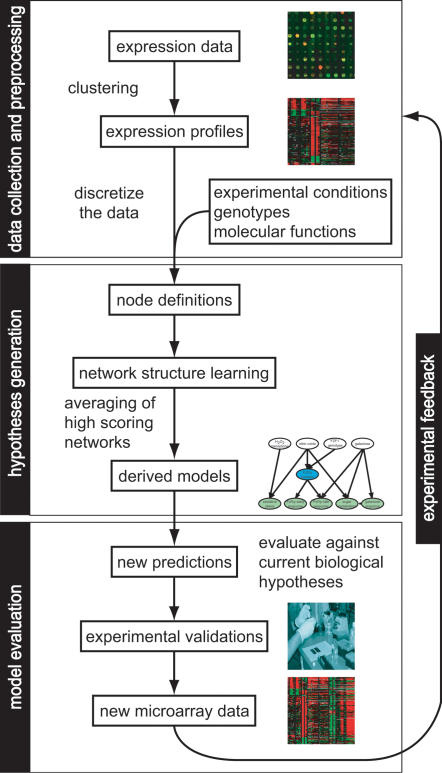
Illustration of the iterative network learning and experimental feedback algorithm.

Our approach iterated through four steps: data collection and preprocessing, hypothesis generation, model evaluation and experimental feedback (Figure 1).

In the data collection and preprocessing step, gene expression clusters were identified from a microarray dataset and cluster expression levels were converted to discrete values. Each array was annotated with the strain genotypes, the experimental conditions, and protein functions. The discrete values of cluster expression and the annotated array attributes were combined to form the learning dataset.

In the subsequent hypothesis generation step, expression clusters, environmental signals and genotypes were defined as network nodes. An automatic learning procedure was used to find network connections that best fit the learning data set, measured by a probability score. Networks with the highest scores were collected. The derived model (Bayesian average network) was the average over all the high scoring networks found by the learning process (Materials and Methods). In the derived Bayesian average network model, each edge was associated with a confidence score (c), calculated as the percentage of its presence in the high scoring collection [18], [21]. As part of the learning process, the conditional probability distribution (CPD) for each node was also automatically inferred.

In the third step, the derived model was compared to the current biological hypotheses and new predictions were then tested experimentally. In the last experimental feedback step, the new experimental data were compared to the data underlying the previous model. If the new data was inconsistent with the previous model, new network nodes such as a new environmental variable or an alternate gene clustering was proposed to attempt to explain the discrepancy. Experiments could also show the new predictions to be incorrect. In either case, we initiated a new iteration of the process to derive a better model, likely with a revised set of network nodes to 1) explain the conflict in the data, 2) predict the role of the new environmental variables on gene expression, and 3) eliminate any incorrect prediction.

In the following sections, we describe three iterations of the algorithm applied to a microarray dataset measuring the nitric oxide transcriptional response, generating increasingly improved transcription network models.

### The initial nitric oxide response network

In order to measure the *S. cerevisiae* transcriptional response to NO· and reactive nitrogen intermediates, and to examine the role of the transcription factor Fzf1p, we exposed wild type and *fzf1Δ* strains to chemically generated NO· (experiment E1, Materials and Methods). To determine whether Fzf1p over-expression could mimic the NO· inducible response, we performed similar experiments with wild type and *GAL1p:FZF1* strains on galactose. We then measured global mRNA levels over time using DNA microarrays (experiment E2, Materials and Methods). These data were combined with a published dataset from a perturbation experiment of yeast treated with common oxidative agents to model the oxidative or environmental stress response (ESR) [9].

A subset of 130 genes with significant expression changes in the combined dataset was selected (Materials and Methods). We defined five major gene clusters: the previously identified detoxification gene clusters [8] were divided into Fzf1p early and late response clusters (which were up-regulated by NO· in an Fzf1p dependent manner, but differed in their initial response time), the ESR cluster, the oxidative phosphorylation cluster, and the galactose response cluster.

We subsequently defined ten network nodes and their discrete states to model the transcriptional response microarray data, which included five gene cluster nodes, three experimental perturbation nodes, one genotype node, and one protein function node, to model the genome-wide transcriptional response to nitric oxide (Table 1).

Given those defined nodes, the learning dataset was compiled by combining discrete values of average cluster expression and manual annotation of experimental attributes for each array. Among them, the values of Fzf1p activity in the learning dataset were inferred based on *FZF1* genotype and the experimental conditions. For example, if the strain was *fzf1Δ*, the value was assigned to “inactive”. Any value that could not be inferred or obtained directly was set as missing values (empty data entries) in the learning dataset.

**Figure 2:**
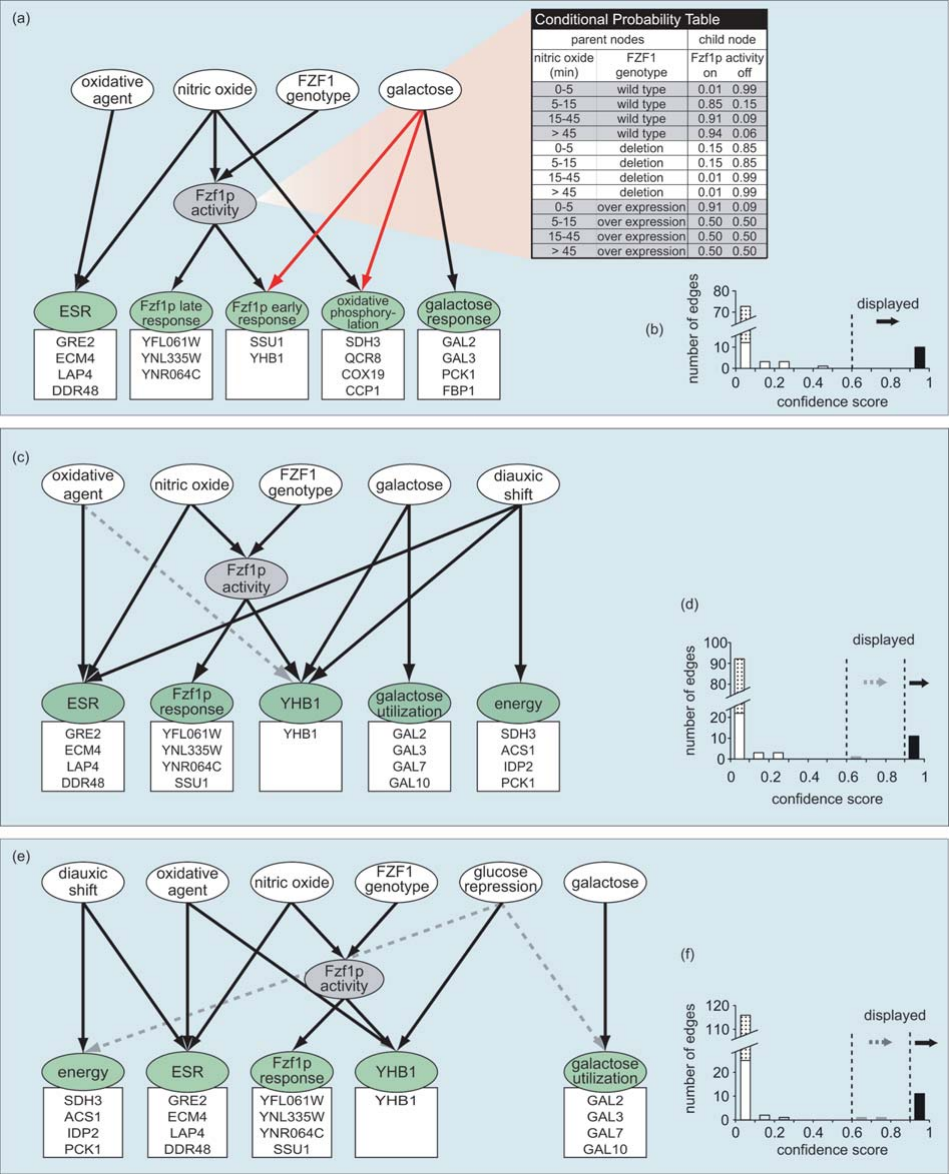
The Bayesian average network representation of the models. (a, b) initial model. (c, d) second model. (e, f) third model. (a, c, e) network graphic representation. The green nodes represent gene expression clusters. Representative genes of each cluster are shown in the box below each node. ESR: environmental stress response cluster. energy: glucose metabolism cluster. oxidative stress: the application of H_2_O_2_ or menadione. Nitric oxide: the duration of NO· exposure. galactose: galactose utilization. diauxic shift: shift between anaerobic growth and aerobic respiration. Nodes with missing values are colored in gray. The CPD table shows the conditional probability distribution of Fzf1p activity. The red edges represent novel predictions from the first network model. (b, d, f) edge confidence score histogram. The dot-filled columns represent edges excluded from a model by structural constraints.

The initial derived model (10 edges with c>0.9) is shown in Figure 2a. According to this model, exposure to NO· generated two transcription signatures. The core NO· specific response (Fzf1p early and late response clusters), unlike other transcriptional responses, was controlled through the activation of the transcription factor Fzf1p. NO· also triggered a general environmental stress response, which was shared by exposing to oxidative agents. Those predictions were consistent with the current understanding of the transcriptional response to NO· [8].

The edge confidence scores (c) displayed a bimodal distribution in the confidence score histogram (Figure 2b). Since the confidence score was a measurement of the data support for an edge, this distribution showed a clear separation of relationships that were highly supported (c>0.9) or unsupported (c = 0) by the data. This bimodal distribution was significantly different (Kolmogorov-Smirnov normality test; *P*<0.001) from the normal distribution generated from networks with the randomly assigned network connections (Kolmogorov-Smirnov normality test; *P* = 0.35), in which all edges showed a low level of support from the data (c = 0.395±0.105).

As part of the network learning process, conditional probability distributions (CPDs) were also computed from the data (supplemental data). This included a CPD of the node “Fzf1p activity”, which had 80% missing values in the learning dataset. The derived CPD of “Fzf1p activity” (the chance of Fzf1p activity in either “active” or “inactive” state given the *FZF1* genotype and the duration of NO· treatment) predicted that Fzf1p activity was transiently activated by NO· treatment (Figure 2a CPD table). The prediction was consistent with the biological hypothesis that NO· or NO· derivatives activates Fzf1p leading to transcriptional induction of a discrete set of target genes that function to protect the cell from NO-mediated stress [8].

**Figure 3:**
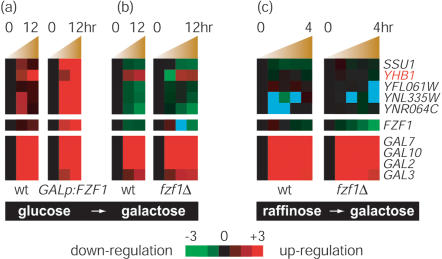
The change of gene expression in Fzf1p response clusters, *FZF1* and galactose utilization genes in response to galactose. (a) Wild type and gal promoter driven *FZF1* over-expression strains in response to the change from glucose to galactose (experiment E2). (b) Wild type and *fzf1*Δ strains in response to the change from glucose to galactose (experiment E3). (c) Wild type and *fzf1*Δ strains in response to the change from raffinose to galactose (experiment E4). Color unit is fold change of gene expression. Gene expression too low to be detected is colored in blue.

Two highly supported edges (c>0.9) in the model were unexpected. One was directed from “galactose” to “Fzf1p early response” and the other from “galactose” to “oxidative phosphorylation” (Figure 2a, red edges). The connection from galactose to Fzf1p early response node predicted that the expression of this cluster (containing *YHB1* and *SSU1)* would be up-regulated in response to galactose (as predicted in the probability distribution for the early response node), suggesting another input signal to some of the NO· detoxification genes bypassing the transcription factor Fzf1p. Further examination of the microarray data showed that *YHB1* was up-regulated by galactose in the absence of Fzf1p over-expression (Figure 3a). *FZF1* levels in wild type yeast were not affected by growth in galactose media. Although our model predicted an Fzf1p-independent up-regulation of the *YHB1* by galactose, it remained a formal possibility that galactose was acting through endogenous Fzf1p to up-regulate *YHB1*.

### Experimental feedback and a second nitric oxide response network

To verify the unexpected *YHB1* induction in response to galactose and the independence of this relationship on Fzf1p, additional microarray experiments were performed to monitor the change of mRNA level upon galactose induction in wild type and *fzf1Δ* strains (experiment E3). Indeed, the expression of *YHB1* was increased by 2–4 fold upon switching to a galactose-containing medium (Figure 3b). This confirmed the prediction that galactose affects *YHB1* expression independently of Fzf1p.

However, we detected an inconsistency in the dataset. The two galactose induction experiments (experiment E2 vs. E3) were conducted in an experimentally similar way, yet many genes which were up-regulated in one experiment were down-regulated in the other and vice versa (supplemental data). For example, the genes in the Fzf1p response cluster (except *YHB1*) were up-regulated in E2 and down-regulated in E3 (Figure 3a wt vs. 3b wt). In contrast, many galactose utilization genes such as *GAL2, GAL3, GAL7* and *GAL10* showed consistent up-regulation in all the galactose induction experiments (Figure 3a & 3b). Most of the genes with between-experiment disagreement function to utilize glucose, such as all four subunits of succinate dehydrogenase tetramer *SDH*, acetyl-coA synthetase *ACS1*, and the key gluconeogenic enzymes *FBP1* and *PCK1*. The opposing expression change (E2 vs. E3, wild type) in these glycolysis and gluconeogenesis components were also highly correlated with their transcription profiles during the diauxic shift, the switch from anaerobic growth to aerobic respiration upon depletion of glucose [9], [22]. An examination of the pre-experimental growth conditions, the cell densities during the experiment, and the duration of the experiment (12 hr) indicated that the diauxic shift could have occurred in the two galactose induction experiments (E2, E3).

Taking advantage of the above prior biological knowledge on the yeast diauxic shift, we added another environmental perturbation node (“diauxic shift”). In addition, we improved the gene clustering by 1) combining the previous oxidative phosphorylation cluster and the galactose response cluster to form the energy cluster; 2) re-grouping the five NO· detoxification genes into *YHB1* and the Fzf1p response cluster that included the rest of the detoxification genes; and 3) separating out the galactose utilization genes to form the galactose utilization cluster (Table 2).

The second Bayesian network model was expanded to take into account the transcriptional response to the diauxic shift (Figure 2c). The new model maintained the sub-network of the NO·-specific response mediated by Fzf1p and the relationship directed from galactose to *YHB1*. In addition, it revealed the previously hidden connection between the diauxic shift and the energy cluster.

### Glucose derepression regulating YHB1 and the third nitric oxide response network

In order to avoid complications due to the diauxic shift in the galactose induction experiments (12 hr), the galactose induction experiments using wild type and *fzf1Δ* strains (experiment E3) were repeated using raffinose as the initial sugar source (experiment E4). This allowed a much faster induction and a shorter time course (4 hr). The results showed that the galactose utilization genes such as *GAL7* and *GAL10* were up-regulated; however, *YHB1* induction was not observed (Figure 3c). This result was unexpected since the previous galactose induction experiments had shown that *YHB1* was induced by 2–4 fold (Figure 3a wt, 3b). The difference could not be explained by the diauxic shift or other variables considered thus far.

Growth in glucose-rich media represses the transcription of a large number of genes such as enzymes in the TCA cycle, the respiratory chain, sporulation genes and genes needed for the utilization of less efficient sugar sources such as galactose [23]. To address the possibility that *YHB1* is partially controlled by glucose repression, a node “glucose repression” was added to account for this effect in a third model (Figure 2e, Table 3). The subsequently derived third model strongly supported the relationship between glucose derepression and *YHB1* gene expression; at the same time the overall network structure including the Fzf1p mediated NO·-specific response sub-network was maintained. The CPD of node “YHB1” predicted that *YHB1* gene expression was up-regulated by either glucose derepression or Fzf1p, but not by galactose.

**Figure 4:**
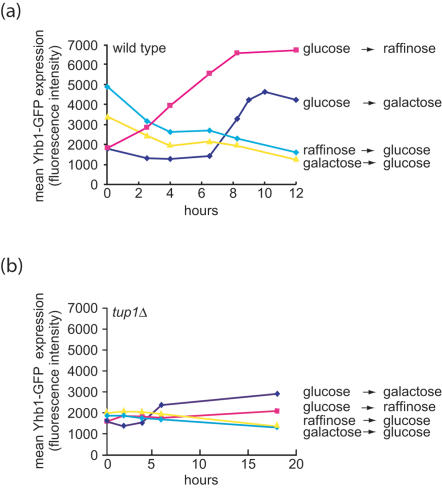
Glucose repression and derepression of Yhb1p-GFP measured by flow cytometry. (a) wild type strain. (b) *tup1* deletion strain. To calculate a mean GFP intensity, a minimum 100,000 cells were measured for each time point.

To verify the prediction of glucose derepression for *YHB1*, the protein expression levels of GFP tagged Yhb1p were monitored under glucose repression and derepression conditions using flow cytometry. The repression results showed that Yhb1p levels decreased immediately after the sugar source was changed from either raffinose or galactose to glucose, and continued to decrease up to 2–3 fold after 12 hours. This result was confirmed by the reciprocal experiment of glucose derepression by changing the sugar source from glucose to galactose or raffinose, in which Yhb1p levels increased by 2–4 fold after 12 hours (Figure 4a). The ratio and kinetics of the *YHB1* derepression measured by protein level were consistent with the microarray measurements (Figure 3a, 3b). Glucose repression of *YHB1* was not observed in a *tup1*Δ strain, indicating that the effect of sugar on *YHB1* expression occurred through the canonical glucose repression pathway (Figure 4b).

## Discussion

We have developed a framework to formally couple Bayesian network learning and experimental feedback to model a specific biological response in yeast. We were able to use this integrative approach to achieve two goals. First, we discovered an additional layer of regulation acting upon *YHB1* transcription, a key mediator of nitric oxide defense. Secondly, our approach dissected out specific versus nonspecific responses to NO· and reactive nitrogen intermediate exposure. The core structure of the Fzf1p-dependent NO·-specific response sub-network (nitric oxide, *FZF1* genotype Fzf1p activity, and Fzf1p response clusters) was predicted and maintained throughout the three models. The transcriptional responses to other environmental factors were gradually elucidated by additional iterations of the process.

Previous studies have suggested that *YHB1* is important for the survival of yeast under oxidative and nitrosative stress [28], [29]. Our results show *YHB1* is transcriptionally regulated by both NO· exposure mediated through Fzf1p and glucose repression mediated by Tup1p. Taken together, these data indicate that *YHB1* is regulated by many environmental signals, highlighting the combinatorial control of this gene. While glucose derepression caused a 2 to 3-fold increase in Yhb1p protein levels, studies have shown a 10-fold increase by NO· treatment, suggesting a more prominent role of Yhb1p in NO· detoxification [8].

Within the context of our Bayesian model, we were able to utilize available biological knowledge to systematically explore the response to nitric oxide by the refinement of the random variables used. Clearly, incorporation of prior biological knowledge has the effect that our results will be biased towards our current understanding of the problem. While this fact represents a caveat, all models make assumptions; and the biological knowledge in this case was extremely useful to uncover the underlying relationships. Indeed, prior knowledge in this case can be considered to be a critical property of the process, since proper definition of the random variables used to model the dataset was essential to arrive at a biologically meaningful conclusion.

A common practice in statistical learning is to select one single model that best fits the data. But in many situations, other models also score very well although not necessarily the best. Using a single highest scoring model to derive a biological conclusion is potentially risky. To circumvent this problem, we used the average of all the high scoring networks found by the searching procedure [21]. An added benefit of this approach is that it yields a confidence score associated with each edge connection [18]. The confidence score is especially useful for filtering out low-confidence connections from a complex network, thus simplifying what might otherwise be a confusing network.

Since Bayesian network edges represent statistical instead of causal relationships, it is possible that a derived edge does not represent a direct biological connection. For example, two gene clusters sharing high mutual information would likely be connected. One method to eliminate such connections is to merge those highly correlated clusters into a single node. Additionally, structural constraints may be used to define gene expression nodes as leaf nodes and the environmental variable nodes as root nodes.

Gene clusters were defined through an automatic hierarchical clustering algorithm with manual interventions. Although it is not purely automatic, this step was where we incorporated prior biological knowledge to interpret the gene expression dataset. Therefore it is critical for ensuring the defined gene cluster nodes that truly represent the underlying gene expression profiles.

The computational framework and experimental approach presented here essentially represents a supervised data exploration system. The overall methodology is a straightforward hypothesis-generation, testing, and refinement cycle. However, complex datasets with large numbers of measurements become increasingly difficult to represent and score with regard to a given hypothesis. The creation and use of Bayesian networks, incorporating prior knowledge, allows for systematic scoring of a given hypothesis, and furthermore, provides an opportunity for automatic learning, which in turn can facilitate the discovery of new relationships within the data.
